# An Exploratory Study on Subject-Specific Throwing Arm Strength Responses to a Novel Intra-Abdominal Pressure Belt Worn by Collegiate Baseball Pitchers

**DOI:** 10.3390/sports13040113

**Published:** 2025-04-10

**Authors:** Ryan L. Crotin, Christian Conforti

**Affiliations:** 1 RC13 Sports, Phoenix, AZ 85007, USA; 2 Human Performance Laboratories, Department of Kinesiology, Louisiana Tech University, Ruston, LA 71272, USA; 3 Sports Performance Research Institute New Zealand, Auckland University of Technology, Auckland 1010, New Zealand; 4 Toronto Blue Jays (MLB), Toronto, ON M5S 1V4, Canada; cconfor@alumni.uwo.ca

**Keywords:** ulnar collateral ligament, Tommy John Surgery, shoulder, elbow

## Abstract

Throwing arm injuries in baseball are related to throwing arm weakness. This case study investigated potential arm strength improvements owed to wearing a specialized intra-abdominal pressure (IAP) to prime arm strength prior to simulated competition. The randomized study design involving 13 collegiate pitchers who threw 3 bullpens of 40 pitches with visual encouragement through an LED-integrated radar gun unit wearing their typical belt, an IAP belt at regular length, and the IAP belt with 2 in cinch. A portable dynamometer evaluated throwing arm strength prior to bullpen sessions wearing each belt type to denote strength responses. Participant-specific data presented in this exploratory study indicated potential benefits for increasing throwing arm strength. Overall, higher throwing arm strength scores were seen for the majority athletes when wearing the IAP belt. As a result, this exploratory case study should inspire future research evaluating IAP influences on throwing arm strength, as elevated proximal stabilization of the core creates a new avenue for improved throwing arm function among baseball pitchers.

## 1. Introduction

Nearly 3 billion dollars has been spent on pitching injuries over the past 20 years in Major League Baseball, mainly attributed to Tommy John Surgery, where a greater economic burden exists amongst amateurs [1]. Current epidemiology indicates that 5 pitchers in every 100 require surgery, and that the percentage of surgical cases has risen amongst collegiate demographics [1,2]. Northern climates are typically known to be safer, at least from an ulnar collateral ligament (UCL) reconstruction perspective, yet some orthopedic centers are performing over 400 cases per year, which is equivalent to more than one Tommy John Surgery per day [3,4]. To estimate financial burdens in parallel to previous findings, with over 16 million baseball players participating in sport each year, and an estimated 1.77 M pitchers, almost 89,000 pitchers undergo throwing arm surgeries per year [5]. Financially, the forecasted equivalent in economic burden is estimated to be more than 2.5 billion dollars per year, which includes the cost of surgery, physical therapy, lost fees paid by athletes and parents to travel should injuries happen in the season, and sadly, psychological counselling for post-traumatic stress disorder and a host of other mental health issues [6]. At present, further risk and greater financial losses may be heightened by a pitch clock strategy instituted by college and Major League Baseball to expedite completion of games [7]. Because of pitch clock integration and reduced rest periods, neuromechanical modeling estimates considerable throwing arm strength loss in the forearm flexor pronator mass muscles, while the current literature indicates that most professional pitchers demonstrate relatively weak throwing arms, exposing them to greater risk [7,8].

Previous work has shown the importance of bidirectional energy transfer through the pelvis, which transfers energy to the trunk as well as the lead leg in its effort to absorb ground contact and stabilize the pitcher for transverse rotation [9]. This relationship is associated with stabilization of the proximal body to prevent early opening upon foot contact and may have deleterious effects on the throwing arm, with altered energy flows [9]. A key component in stabilizing the proximal chain to assist the trunk to be more closed at foot contact involves coordinated contraction of five specific muscles; the internal obliques, quadratus lumborum, transverse abdominus, rectus abdominis, and external obliques that regulate trunk motion [9]. The proximal chain is also cylindrical and hollow, which provides a large intra-abdominal cavity and stabilizing air bladder in the stomach, as an adjunct to improved compression and torsional load resistance in the spine [10]. With engagement of the core musculature, diaphragm, and paraspinal muscles, the proximal cavity becomes ridged through increased intra-abdominal pressure (IAP) that supports the spine for rotation and improves force generation by distal segments of the upper and lower hemispheres of the body. Shoulder and elbow weakness is exacerbated by altered proximal motor patterns affecting functional joint stabilization; for example, a change in stride strategy can lower grip function [11]. With the noted impact of proximal influences on throwing arm strength, it is possible that improved co-contraction of core muscles to raise IAP may elicit greater throwing arm strength in pitchers, which may minimize throwing arm injury risks associated with shoulder and elbow muscle weakness causing loss of joint stability.

Prediction of throwing arm injuries largely focuses on three-dimensional motion capture in determining factors that increase throwing arm forces; however, it does not indicate information on altered throwing arm strength capacity and is not a feasible surveillance option for in-game competition across all levels of play [12,13]. Other work has shown strength deficits arising from altered 3D mechanics, which provides an opportunity to examine throwing arm function more ubiquitously and can potentially lessen injury risk by strengthening the throwing arm. Therefore, this study aims to examine the performance of a unique baseball belt designed with the intention of increasing functional intra-abdominal pressure (IAP) that can be worn innocuously in games and to evaluate its impact on throwing arm strength in pitchers. More specifically, this exploratory case study focuses on identifying the participant-specific impacts of wearing a novel intra-abdominal pressure belt on relative throwing arm strength and three-finger grip strength primarily targeting the function of the flexor digitorum superficialis (FDS) muscle in high-level pitchers. It is hypothesized that increased intra-abdominal pressure arising from a stronger and tighter baseball belt will increase global throwing arm strength-to-body mass ratios and absolute FDS strength capacity among individuals prior to participating in maximum-effort pitching events. Given positive results, this case study can be expanded toward larger research undertakings that raise the protective value of baseball belts through studies associating IAP and improved neuromuscular function that raises throwing arm strength and augments dynamic stabilization in absorbing high rates of joint loading.

## 2. Methods

**Figure 1:**
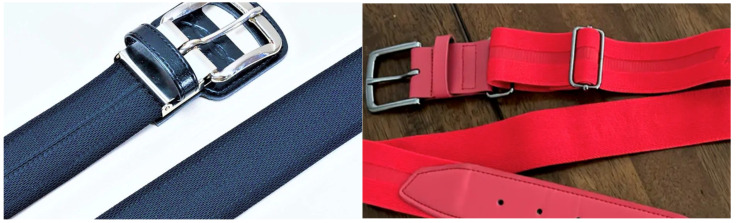
Image of the intra-abdominal pressure belt and typical belt worn in this case study. The intra-abdominal pressure belt worn in this study (**left image**) was 2 mm thicker than the typical elastic baseball belt (**right image**) worn by collegiate baseball players playing the United States.

The research design involved a case study of 13 collegiate pitchers (height; 1.86 ± 0.06 m, body mass; 88.5 ± 8.31 kg, age; 20.6 ± 1.39 years) who signed an informed consent that communicated the risks and benefits associated with their participation. Participants were recruited from one collegiate team and tested over 3.5 weeks with no obstruction or interference from team activities with the aim of lessening the influence of individual throwing arm fatigue, which could have arisen from an open recruitment process with players exerting themselves differently based on varying practice schedules, Fall competitive seasons, or training programs. Dietary and training records were not requested in this study, and throwing programs were not monitored between testing sessions; however, rest guidelines between testing sessions were provided in the informed consent and communicated consistently to participants prior to and during the study. All athletes had to be medically cleared by their respective college program to volunteer. This study was approved by the Institutional Review Board at Arizona Christian University, which approves research in accordance with the requirements of the Declaration of Helsinki Act for the ethical principles of research involving human subjects (IRB21CFR56.108). The comparative analysis involved players wearing a regular baseball belt with a thickness of approximately 1 mm, while the IAP belt maintained a thickness of 3 mm when worn with baseball pants (which were unchanged) cinching the belt two inches from regular length (see Figure 1). In the sport of baseball, players wear baseball belts with their pants during competition. Therefore, a controlled study without the use of a baseball belt would not be applicable to real-life conditions. All participants were first introduced to the IAP belt during the time of this study. Functional strength assessments were undertaken prior to all bullpens and conducted using a portable dynamometer and a companion password-encrypted smartphone application that stored and visualized data which could then be further examined on a password-encrypted dashboard that consolidated throwing arm strength metrics to be visualized on a computer or downloaded in CSV format for later analyses (ArmCare.com, Indialantic, FL, USA). The dynamometer unit (ActivForce^®^, San Diego, CA, USA) which is compatible with the smartphone application (ArmCare.com^®^, Indialantic, FL, USA) was mechanically tested for calibration accuracy, and this revealed strong agreement until 45 kg (99 lbs of force), which is well over the strength capacity of any individual strength measure for the throwing arm musculature. Testing results can be seen in Table 1.

Mechanical testing was undertaken by Hardworks Inc. (Ichalkaranji, India. Hardworks.com). The table above references differences with respect to the values measured by the portable dynamometer used in this study. Positive numbers indicate greater differences for the portable dynamometer, while negative differences indicate lesser force values compared to mechanical force readings. The portable dynamometer demonstrated suitable accuracy up to 45 kg (less than 1.5 lbs difference), which is beyond any human strength performance achieved for upper extremity test protocols.

**Figure 2:**
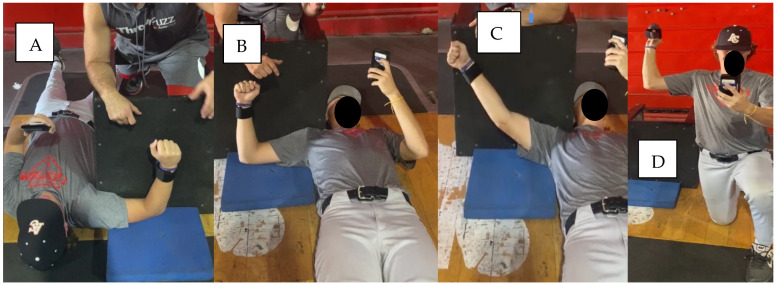
Order of portable dynamometry strength tests for the throwing arm and brief functional purpose of each test. Throwing arm strength tests were performed supine and in half-kneeling positions and guided by a smartphone application for shoulder and elbow strength tests. A three-finger grip strength test was instituted to pinpoint the strength performance of the flexor digitorum superficialis, a main forearm stabilizer protecting the ulnar collateral ligament from tensile stress. Testing collage: (**A**), maximum internal rotation strength measurement; (**B**), maximum external rotation strength measurement; (**C**), maximum co-contraction strength measurement of the rotator cuff muscles and deltoid; (**D**), maximum strength measurement for the flexor digitorum superficialis. All muscles tested have biomechanical relevance to the throwing delivery.

Strength testing occurred prior to all bullpen sessions and commenced with a 6-min, standardized, guided warm-up using bands (Crossover Symmetry Inc., Indialantic, FL, USA), which was visualized with video integration on the smartphone application. As an overview, after the warm-up was completed, athletes wore a wearable dynamometer around their wrist for shoulder strength testing and held the dynamometer in a free-standing position, grasping the device in a three-finger grip as if holding a baseball in a half-kneeling position. Specifically, internal, external, and scaption strength testing required athletes to make a tight fist while pushing the wearable dynamometer maximally into an immovable plyobox in a supine position with the legs outstretched to reduce muscle irradiation effects on the lower extremities (i.e., pushing on the floor to increase the push force of the upper limb). After completing all shoulder tests, the athlete positioned himself in a half-kneeling position to perform the baseball grip strength test in 90-degree shoulder abduction, external rotation, and elbow flexion positions. Functionally, the internal rotation test evaluates the strength of the muscles that accelerate the arm forward and decelerate it as it goes backward. The external rotation test evaluates the strength of the muscles that primarily decelerate the throwing arm after ball release and accelerate the arm into the layback position. The scaption test evaluates strength in an outstretched arm position, which reveals the capacity for integral force at the point at which the ball is released; the three-finger baseball grip test evaluates strength of the forearm muscles involved in stabilizing the elbow and finger flexion force, which applies friction to the ball through the index, middle finger, and thumb. A countdown of 10 s was provided through the smartphone application prior to the athlete providing a maximal isometric pressing force. When testing, athletes provided two maximum effort repetitions separated by 10 s to increase intermittent recovery demand on the neurological system, where each repetition consisted of a 3 s isometric contraction. If consecutive force readings were not within a 10% coefficient of variation, a third repetition was initiated. Max performance, indicated by the highest neuromuscular output, was stored in the cloud and exported to a CSV file for participant-specific interpretations. For consistency, athletes did not receive verbal encouragement from examiners during testing, yet visual reinforcement was provided by the app and was indicated by a digital strength dial to motivate the athlete to push with maximal isometric force. Following strength assessment, athletes were cleared to start their throwing warm-up. Full visual demonstrations and a brief description of the functional purpose of each strength test can be seen in Figure 2.

Following throwing arm strength assessment, pitchers threw three separate bullpen sessions involving two uniform belts. Prior to bullpen testing sessions, athletes utilized their self-selected throwing warm-up routine and began their simulated game when verbally ready to throw at maximal effort. A randomized table of numbers organized athletes and assigned their order of pitching conditions. Athletes pitched bullpen sessions of 40 pitches from an indoor mound, separated by two innings of 20 pitches with a 5-min intermission between innings using a regular elastic baseball belt mandated by the collegiate team (REB) that does not tighten around the lower abdominal area, which is a more rigid intra-abdominal pressure belt (IAPB) (Core Energy Belt^®^, Core Technology Inc., Irvine, CA, USA). This belt had the same length as their typical belt and provides more support and is less elastic around the lower abdominal area; athletes also wore an intra-abdominal pressure belt cinched two inches tighter than their typical belt size to maximize intra-abdominal pressure (IAP2) (Core Technology Inc., USA). Extreme tightening, stronger material properties, and the added thickness of the intra-abdominal belt in the IAP2 condition created a more robust retaining wall around the lower abdomen, which can accentuate core co-contraction beyond other belt conditions in the study. As a result, the coordinated contraction of the intrinsic core muscles, paraspinals, and diaphragm pushing against the belt offered the highest IAP condition.

Bullpens were designed to simulate games with pitch velocity and location tracking. Each pitcher threw 2 fastballs-to-1 change up until all 40 pitches were thrown, with a 15 s rest between each pitch thrown. Prior to the first simulated innings, each pitcher was provided eight pitches to warm up while on the portable mound and five pitches prior to the second innings to prepare for maximum-effort throwing. In total, each pitcher threw 159 pitches across the three bullpen sessions. A minimum of 72 h rest was provided between bullpen sessions.

Velocity was tracked using a widely used portable radar unit and LED screen that provided visual feedback to the athlete to encourage high-intent throwing in delivering the baseball (Pocket Radar^®^, Santa Rosa, CA, USA). Velocity records have been published in a previous study and reported elsewhere [14].

### Statistical Analyses

**Figure 3:**
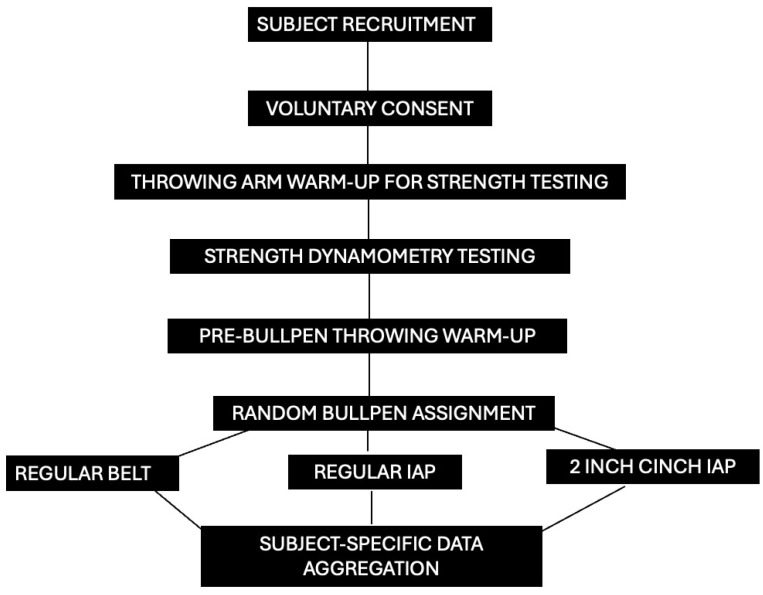
Full study design flow chart. Regular IAP: intra-abdominal pressure belt bullpen conditions with regular belt length 2 in Cinch IAP: intra-abdominal pressure belt bullpen conditions when the athlete is wearing the IAP belt two inches tighter than the regular length of the belt.

After the completion of all bullpens thrown, data were exported to a CSV file (Excel^®^, Microsoft, Redmond, WA, USA) and analyzed for group- and participant-specific differences in overall relative throwing arm strength. Descriptive statistics that include means, standard deviations, 95% confidence intervals were calculated across belt conditions. In previous research, arm strength data were presented as both relative and absolute data, with relative data indicating significant deficits for a population comparable to the study sample in this work [8]. Based on this study featuring dynamometry and high level baseball pitchers, the sample size power analysis indicated that 13 athletes were underpowered to present findings with inferential statistics and effect size calculations. Therefore, case study measures, such as evaluating descriptive and participant-specific differences, were computed to present exploratory, arm strength responses to belt conditions. Overall scores combining internal, external, and grip strength measures provided a comprehensive evaluation of all muscle group contributions to maximum strength and were summarized as the ArmScore. Mathematically, the ArmScore is a conglomerate sum of all the previously mentioned strength scores divided by the body mass of the athlete to depict an overall determination of throwing arm strength relative to the size of the athlete. In previous work, relative strength measures for the throwing arm presented weakness amongst North American professional pitchers and was used as a determinant in this study to indicate the extent of global throwing arm strength responses [8]. Absolute strength in three-finger grip represents the strength of the FDS, a critical dynamic stabilizer of the UCL that is anatomically situated over the top of the UCL and activated when gripping a baseball. Absolute strength measures were selected for this indicator of dynamic strength in the elbow region, as previous research has demonstrated the stress-shielding effects of absolute strength, which is critical for the medial elbow in protecting the ulnar collateral ligament from elbow varus torque overload [15]. Although the subject pool was underpowered, a Repeated Measures ANOVA with Bonferroni Correction (SPSS 29.0, IBM Corp, Armonk, NY, USA) was undertaken to determine the possibility of significant differences with an a priori of 0.05. Following analysis of variance, effect size calculations (SPSS 29.0, IBM Corp, USA) were undertaken to determine clinically relevant differences in ArmScore and FDS strength measures between conditions. For a complete visualization of the study design, please see Figure 3.

## 3. Results

ArmScore differences were not significant indicating no main effect of condition on relative throwing arm strength. Although strength increases from REGB to RIAP to 2IN were seen with a sizable effect size, significant differences were not seen across belt conditions (F = 2.176, *p* = 0.135, ηp^2^ = 0.154). A significant main effect of condition was found for FDS grip strength with a large effect size (F = 3.709, *p* = 0.039, ηp^2^ = 0.236). However, after the Bonferroni correction was applied in post-hoc, no significant rela-tionships were detected that may be attributable to an underpowered sample size.

However, on an individual level, this exploratory case study revealed that the IAP conditions tended to increase relative strength of the arm and three-finger grip. The IAP belt worn at regular belt length showed slightly more increased strength versus the IAP2 condition with the belt cinched two inches tighter than regular belt length. Participant-specific responses varied by the individual, indicating that belt settings could be individualized per player, as some athletes responded better to a tighter fit and may require greater IAP and belt constriction to augment proximal stiffness and co-contraction involving the core, paraspinal muscles, and diaphragm in order to raise throwing arm force (see Table 2 and Table 3).

Maximum relative throwing arm strength as a percentage of body mass for each participant in association with each belt condition. Bolded numbers indicate the participants’ highest strength values across belt conditions. As shown, 77% of participants indicated greater total throwing arm strength relative to body mass prior to bullpen sessions using an intra-abdominal pressure belt, with the IAP belt at regular length indicating slightly better responses. Large effects were seen, yet no significant differences were detected for main effect of condition. Note: IAP% is the percentage of strength change with highest IAP value compared to the regular baseball belt. Positive values denote improved strength. Belt groups: regular IAP Belt; IAPB, 2-inch cinch IAP Belt; IAP2, and regular belt; REGB, RM ANOVA; Repeated Measures ANOVA.

Maximum three-finger grip strength for the throwing arm of each participant in association with each belt condition is shown. Bolded numbers indicate participants’ highest strength values across belt conditions. As shown, approximately 93% of participants indicated greater total grip strength relative to body mass prior to bullpen sessions using an intra-abdominal pressure belt. More athletes performed better with the IAP belt set to regular length versus a two-inch cinch. Significance was met for main effect of condition, but after Bonferroni correction applied in post-hoc, no significant differences were seen between conditions despite large effect size. IAP% is the percentage of strength change with the highest IAP value compared to the regular baseball belt. Positive values denote improved strength. Belt groups: regular IAP Belt; IAPB, 2-inch cinch IAP Belt; IAP2, and regular belt; REGB. RM ANOVA; Repeated Measures ANOVA.

## 4. Discussion

To assist athletes in coordinating IAP, previous work has studied the influence of powerlifters’ use of weight belts on physical performance [10]. Results have shown greater lower body power with heightened paraspinal muscle recruitment that stiffens the proximal region of the body, allowing distal segments of the lower limb to apply force at a faster rate downstream, which is associated to a more ridged kinetic link [10,16,17]. Despite evidence of elevated lower body performance, a gap in the literature exists as it relates to the degree to which IAP boosts the characteristics of upper extremity force in throwing athletes. Thus, this study is the first exploration evaluating throwing arm strength responses to varying degrees of intra-abdominal pressure applied by a novel baseball belt prior to simulated competition amongst collegiate pitchers. Although significant group differences were not demonstrated, our hypothesis was supported, as participant-specific data revealed that most of the players analyzed saw increased relative throwing arm and maximal FDS strength in wearing an IAP belt versus their traditional belt worn at regular belt length. The data in the current study present positive implications for advanced global throwing arm and regional medial elbow stabilizing strength prior to entering competitive events for high-level pitchers, arising from enhanced IAP; the data also show that more effective contraction of the core musculature, the diaphragm, and paraspinal muscles has the potential to promote greater dynamic stability of the proximal body, which could raise the neural activation of the throwing arm [10].

Forward dynamics analyses have indicated that the forearm musculature is critical in shielding the UCL from stress, and therefore, dedicated science efforts must be undertaken to prevent functional forearm fatigue [18]. A greater number of participants in this exploratory work showed increased FDS strength prior to throwing in simulated competition. Elevated FDS strength has positive implications that may advance a players’ ability to sustain dynamic stability and withstand fatigue through the strength-increasing potential of wearing a functional IAP belt that is believed to increase IAP and the co-contraction of the diaphragm, intrinsic core musculature and paraspinal muscle groups. With increased proximal stability across the core region, greater activation can be realized in the distal extremities in order to elicit greater force production and speed application of force to augment contractile strength and power [10]. With continued research involving a larger sample, it is possible that injury risk to the throwing arm could be dramatically mitigated with heightened IAP, as the previous literature has indicated a 36-fold risk of surgical intervention when pitching with a fatigued and weakened throwing arm [19].

Biomechanical compensations occurring below the proximal center of the body can exacerbate throwing arm strength loss [11]. With altered lower body function, either by fatigue or a change in lower extremity joint mobility, sustained throwing arm strength is critical in overcoming potential throwing arm weakness that may lead to a loss of dynamic stability of the shoulder and elbow [11]. In connection to the participant-specific data in this work, it is possible that increased IAP, assisted by a functional IAP belt, could provide injury protection for athletes by maintaining throwing arm strength and lessening strength losses owing to compensatory mechanical adaptations over the course of a season [11,20,21].

The IAP belt concept could be especially important for athletes who have sustained a previous surgical repair. Research indicates that strength requirements significantly elevate amongst injured baseball pitchers as ligament stiffness properties are reduced [22]. The average load acceptance by the UCL is 30 Nm, while a previous surgical repair to the ligament reduces the tensile force resistance to 20 Nm [22]. If the average fastball thrown at 90 mph is 100 Nm, athletes must recruit over 60% of their maximum stabilization strength of the forearm to protect the ligament from failing on every pitch thrown [22]. In the event that an athlete has throwing arm weakness, a key feature previously characterized in pitchers who required UCL reconstruction, the IAP boost offered by a functional baseball belt to overall strength of the arm may prevent injury to the medial elbow with repetitive throwing [20].

Ultimately, the mechanism of injury for the throwing arm arising from throwing arm weakness is its inability to handle loading variability. A recent computational model examined the loading variability on the UCL that arises with dynamic stabilization fatigue, which may overwhelm the ligament and cause it to fail [23]. Baseball has the most games played of all major sports, and pitchers can throw more than 200 innings per season. Given that the average pitcher throws 16 pitches per innings, pitchers can accumulate over 3000 competitive pitches per year, while throws made in non-competition situations are not quantified but can potentially have more catastrophic effects on injury frequency due to altered loading [24]. Based on the computed accumulation of throws and pitches, intuitively varying joint loads will occur, and as a protective strategy, increased throwing arm strength through advanced IAP may heighten force absorption and reinforce the joint to withstand load variance when wearing IAP belts in games and practices [18,23].

The trunk represents the greatest momentum factor in linear-to-rotational momentum exchanges for baseball pitchers [25]. Altered momentum transfer, which is the exchange of segment velocities and their respective mass from heavier to lighter segments, could overwhelm the throwing arm if trunk linear or angular velocities are heightened or early trunk opening occurs [12,13,25]. Traditional weight belts appear to improve proximal intersegmental exchanges in force, momentum, and power for the lower limb, and therefore, momentum exchanges between the trunk and throwing arm could be better regulated by boosting IAP to assist the proximal body in decelerating and positioning the trunk for a more efficient momentum exchange with the throwing arm [10,12,13,25]. Future biomechanical research involving augmented throwing arm strength through heightened IAP may reveal that athletes deliver the baseball with greater velocity relative to joint torques for the shoulder and elbow [9,26]. Ultimately, along with elevating the throwing arm’s dynamic strength qualities in priming it prior to competition, future studies illustrating the degree to which IAP contributes to a more efficient delivery may increase the likelihood of lessening neurological effort for the pitcher, which is expected to provide significant benefits in sustaining throwing arm strength across a competitive season [11,26].

### Limitations

Although this study has shown the positive implications of wearing a specialized belt designed to increase IAP, there are some clear points to consider when interpreting results which mean that the benefits cannot be generalized to all baseball pitchers. This study was an exploratory case study evaluating increasing throwing arm strength with differing IAP levels. Thus, the study had the potential to be underpowered, as no reference study existed examining IAP’s impacts on throwing arm strength in throwing athletes with which we could compare for power analysis. Given the exploratory nature of this work, normalcy testing was not integrated, and coupled with a small sample (N = 13), this meant that the likelihood of a Type II error (failing to detect a true effect) was high; therefore, to evaluate significant findings beyond participant-specific responses, a larger sample size in future would be needed to determine the true effects of wearing an IAP belt. Secondly, comparison of cinching the regular belt, which is highly elastic, would allow additional insights if the traditional belt on its own provided IAP support when tightened maximally to express similar findings. Given this study was the first time athletes had worn the IAP belt, it is possible that a placebo effect was present, as some athletes could have subconsciously believed that the IAP belt would perform better than their regular team belt. Workload and training between testing sessions could not be controlled for collegiate athletes who had to maintain pitching, throwing, and lifting schedules as mandated by the team and were also given self-selected opportunities to program themselves between testing sessions. As a result, workload variability may have been quite high between participants in this small subject pool, which may act as a covariant impacting the analysis of participant-specific responses. Other interactions that may have been present could be a variety of physiological changes and training influences that may have occurred during the study, including dehydration, micronutrient deficiencies, hormonal expression, or other anthropometric effects impacting strength levels that may have arisen from lean mass, body fat gains, or the potential of increased height. Another limitation, one that is common to all laboratory study, is the absence of real game conditions. Batters, clay mounds, and cleats were not involved in this work and may have resulted in different conclusions. Although the realism of actual competition was not offered in the study design, simulated bullpen sessions did encourage elevated adrenaline as velocity feedback was visualized on each pitch to inspire maximum effort. The results of the study may have differed had we used more invasive testing protocols to quantify actual IAP levels for each individual athlete. As a result, IAP may have been greater for some individuals than others despite standard belt settings. In future, IAP quantification for each athlete could further results to associate levels of throwing arm strength with calculated levels of IAP. It is also important to note that the individual results of some participants who reported increased arm strength may be due to individual differences, such as arm segment anthropometrics or training level, and not necessarily to the effect of the IAP belt itself. In future, controlling for these covariates may produce clearer performance measures as a result of wearing the IAP baseball belt; ultimately, expanding this case study to a larger sample size is recommended to demonstrate differences in both statistical significance and effect size. Although the results suggest that further, more controlled studies are needed to fully understand the overall effect of IAP on throwing arm strength, this exploratory work revealed the potential of raising throwing arm strength with a belt designed to increase IAP. In practical applications, a rise in throwing arm strength with abdominal wall constriction could increase ball velocity, pitch deception, and throwing accuracy [14]. Therefore, raising IAP beyond levels achieved by a typical baseball belt can aid in priming the throwing arm before competition, which may improve performance in high-level pitchers.

## 5. Conclusions

Participant-specific throwing arm strength responses indicated priming effects for the throwing arm prior to simulated competition for a majority of college pitchers in response to a novel baseball belt that is designed to raise intra-abdominal pressure. Thus, this exploratory case study supports the need to advance research on throwing arm function through evaluating the interaction between increased proximal stiffness arising from elevated IAP and throwing arm strength responses prior to high-intensity throwing bouts.

## Figures and Tables

**Table 1:** Mechanical calibration testing of the portable dynamometer.

	Actual	Measured	Difference
Tare	2.40	2.00	−0.40
5	7.41	6.9	−0.51
10	12.44	11.9	−0.54
15	17.5	17.2	−0.30
20	22.48	22.2	−0.28
25	27.50	28.0	0.50
30	32.51	33.2	0.69
35	37.53	38.2	0.67
40	42.54	43.0	0.46
45	47.54	48.7	1.16

**Table 2:** Participant-specific and group effects data indicating relative throwing arm strength performance in association with belt conditions.

Player ID	REGB (%BW)	RIAP (%BW)	2IN (%BW)	IAP%
1	70.1	76.8	**96.4**	**37.5**
2	**89.4**	**89.4**	78.5	0
3	77.4	71.6	**83.1**	**7.36**
4	85.2	**90.1**	89.9	**5.75**
5	**109.2**	101.4	106.3	−2.66
6	104.3	**119.5**	113.7	**14.6**
7	70.7	**100.9**	91.7	**42.7**
8	88.4	**101.8**	92.2	**15.2**
9	82.6	79.1	**95.2**	**15.3**
10	**92.0**	87.9	74.2	−4.46
11	79.3	**84.7**	80.2	**6.81**
12	61.9	56.7	**74.1**	**19.7**
13	70.1	76.8	**96.4**	**37.5**
Mean	83.1	87.4	90.1	15.0
Std Dev	13.8	16.1	12.0	15.6
95% CI	75.6, 90.6	78.7, 96.2	83.6, 96.7	
RM ANOVA	F = 2.18	*p* = 0.001	ηp^2^	0.15

**Table 3:** Participant-specific and group effects data indicating absolute baseball grip (three-finger grip) strength performance in association with belt conditions.

Player ID	REGB	IAPB	IAP2	IAP%
1	30.1	32.7	**35.8**	**18.9**
2	33	**48.8**	43.7	**47.9**
3	48.1	**55.9**	46.5	**16.2**
4	28.2	**29.2**	21.4	**3.54**
5	**37**	33.9	31.1	−8.38
6	24.4	**26.7**	25.2	**9.43**
7	39.8	41.3	**42.3**	**6.28**
8	44	**47.3**	31.3	**7.5**
9	31.1	33.8	**36.1**	**16.1**
10	34.6	**41.9**	31	**21.1**
11	32.1	31.7	**32.5**	**1.25**
12	40.9	**43.7**	38.8	**6.85**
13	31.4	31.6	**32.6**	**3.82**
Mean	35.0	38.3	34.5	11.6
Std Dev	6.7	8.8	7.1	13.6
95% CI	31.3, 38.6	33.6, 43.1	30.6, 38.4	
RM ANOVA	F = 3.71	*p* = 0.04	ηp^2^	0.24

## Data Availability

Data supporting reported results are not available publicly due to privacy or ethical restrictions.
